# Microstructure and Genetic Polymorphisms: Role in Motor Rehabilitation After Subcortical Stroke

**DOI:** 10.3389/fnagi.2022.813756

**Published:** 2022-02-01

**Authors:** Jingchun Liu, Caihong Wang

**Affiliations:** ^1^Department of Radiology and Tianjin Key Laboratory of Functional Imaging, Tianjin Medical University General Hospital, Tianjin, China; ^2^Department of MRI, Key Laboratory for Functional Magnetic Resonance Imaging and Molecular Imaging of Henan Province, The First Affiliated Hospital of Zhengzhou University, Zhengzhou, China

**Keywords:** cerebral infarction, corticospinal tract (CST), genetic polymorphisms, motor, rehabilitation

## Abstract

**Background and Purpose:** Motor deficits are the most common disability after stroke, and early prediction of motor outcomes is critical for guiding the choice of early interventions. Two main factors that may impact the response to rehabilitation are variations in the microstructure of the affected corticospinal tract (CST) and genetic polymorphisms in brain-derived neurotrophic factor (BDNF). The purpose of this article was to review the role of these factors in stroke recovery, which will be useful for constructing a predictive model of rehabilitation outcomes.

**Summary of Review:** We review the microstructure of the CST, including its origins in the primary motor area (M1), primary sensory area (S1), premotor cortex (PMC), and supplementary motor area (SMA). Damage to these fibers is disease-causing and can directly affect rehabilitation after subcortical stroke. BDNF polymorphisms are not disease-causing but can indirectly affect neuroplasticity and thus motor recovery. Both factors are known to be correlated with motor recovery. Further work is needed using large longitudinal patient samples and animal experiments to better establish the role of these two factors in stroke rehabilitation.

**Conclusions:** Microstructure and genetic polymorphisms should be considered possible predictors or covariates in studies investigating motor recovery after subcortical stroke. Future predictive models of stroke recovery will likely include a combination of structural and genetic factors to allow precise individualization of stroke rehabilitation strategies.

## Introduction

Most stroke patients experience motor deficits (Johnson and Westlake, 2021) that adversely affect clinical outcomes and impair activities of daily living (Patel et al., 2020). The early prediction of motor outcomes is of great significance for improving prognosis and designing interventions. Many factors impact motor rehabilitation, including individual factors (e.g., age and sex) and disease severity (e.g., lesion location, lesion size, and white-matter tract integrity; Sterr et al., 2014). However, these traditional factors have many limitations in predicting rehabilitation outcomes following subcortical stroke. The white-matter tract integrity of motor pathway in traditional factors is an important factor in directly assessing the prognosis of patient motor function (Liu et al., 2020). However, there are still some challenges in using a single white-matter tract integrity indicator to predict the recovery of motor function in clinical applications. Increasing evidence suggests that genetic polymorphisms may partially explain the frequently reported variability in individual responses to interventions for rehabilitation (Shiner et al., 2016). However, the relationship between genetic polymorphisms and the degree of motor rehabilitation is complex, therefore the single genetic factor also cannot comprehensively predict rehabilitation outcomes after subcortical stroke. Currently, several studies have found that a predictive model combining the microstructural integrity of the motor pathway in traditional factors and gene polymorphisms is significantly better than for models with only one factor for stroke patients (Chang et al., 2017).

The integrity of motor pathways (e.g., the corticospinal and cortico-rubro-spinal tracts) plays an important role in motor dysfunction and recovery after stroke (Kim et al., 2018; Guo et al., 2019; Zolkefley et al., 2021). The corticospinal tract (CST) is the most important motor output pathway and has been deemed the most common locus of motor deficits in subcortical stroke. CST fibers arise from the primary motor area (M1), the premotor cortex (PMC), the supplementary motor area (SMA), and the primary somatosensory area (S1; Schieber, 2007; Welniarz et al., 2017). Differential involvement of the CST plays an important role in determining the outcomes of motor rehabilitation following subcortical stroke.

Genetic polymorphisms that can affect neuroplasticity include those of brain-derived neurotrophic factor (BDNF), the dopamine neurotransmitter system, and apolipoprotein E (Stewart and Cramer, 2017). Since BDNF is the most important neurotrophin, BDNF polymorphisms play a key role in neuroprotection and motor recovery after subcortical stroke (Berretta et al., 2014; Balkaya and Cho, 2019).

The purpose of this article was to review the role of CST microstructure and BDNF polymorphisms in motor recovery after subcortical stroke, which will be useful for constructing predictive models of rehabilitation outcomes and thus for precise individualization of rehabilitation strategies.

## Microstructure of The CST Affects Motor Recovery

The CST is the most important motor output tract, and studies have found that its impairment affects motor rehabilitation (Schaechter et al., 2009; Radlinska et al., 2010). The CST can be reconstructed using diffusion tensor tractography (Mori and van Zijl, 2002), which can visualize the spatial relationship between the CST and infarct sites and the extent of CST damage. Studies have shown that if the CST is mostly or completely impaired by the infarct, nerve fiber tract damage is more serious, usually with partial or complete interruption, which correlates with poor recovery of motor function after treatment. In patients with infarcts near the CST, nerve fiber tract compression and deformation but no obvious damage is observed, and good motor function recovery is achieved after treatment. With no obvious relationship between the CST and the lesion, motor function involvement is not observed (Liu et al., 2020). Changes in diffusion tensor imaging (DTI) parameters can be used to observe the spatial relationship between the CST and infarct lesions, and the degree of CST damage observed predicts the prognosis of patients with subcortical stroke. DTI responds to the pathophysiological changes in the infarcted tissue, evaluates the degree of lesion tissue damage, and predicts clinical prognosis. One of the parameters accessed by DTI is fractional anisotropy (FA), a measure of microstructural integrity. A longitudinal study showed that the FA value in the affected CST changes dynamically, decreasing rapidly within 1 month after stroke, slowly decreasing from 1 to 3 months after stroke, and showing little change after three months (Yu et al., 2009). A significant correlation has been reported between FA in the CST and motor outcomes in patients with stroke (Stinear et al., 2007; Xue et al., 2021). Although M1 is thought to be the main origin of CST fibers, CST fibers also arise from the PMC, SMA, and S1 (Schieber, 2007; Welniarz et al., 2017). Considering the functional differences between these brain regions, CST fibers originating from these cortical regions may be responsible for different functions, and impairments of these fibers may result in distinct functional deficits. Therefore, some DTI studies have attempted to reconstruct the trajectories of CST fibers with different origins (Newton et al., 2006; Seo and Jang, 2013).

Considering the CST as a whole tract, cross-sectional studies have shown that decreases in the FA value of the CST (Liu et al., 2015) are related to motor recovery in subcortical stroke (Schaechter et al., 2009). In addition, the CST injury was also evaluated by other methods, involving the percentage of damage of CST fibers (Riley et al., 2011; Liu et al., 2020), raw- and weighted-CST lesion loads (Zhu et al., 2010). Liu et al. further found that the isotropy of M1 fibers was correlated with walking endurance and that of SMA fibers with motor dexterity in healthy adults. They also found that the early damage of CST originating from the M1 and SMA is closely associated with motor outcomes and brain structural changes in chronic stroke patients (Liu et al., 2020). These results suggest that the microstructures of the CST affect motor rehabilitation. Therefore, CST microstructure could be used to predict long-term motor outcomes, which is clinically important because the early prediction of motor outcomes is critical for designing suitable interventions for patients with subcortical stroke. Kim et al. (2021) indicated that CST microstructure was a significant predictor of improvement in chronic stroke survivors with mild-to-moderate motor impairment. Lin et al. (2019) compared the prediction performance of four different CST injury methods and showed that CST injury explained ~20% of the variance in the magnitude of upper extremity recovery even after controlling for the severity of the initial impairment. Moreover, Burke Quinlan et al. evaluated CST injury by probing FA and the percentage of damage of CST fibers. They found that the best prediction in multivariate modeling was achieved using CST injury, and Lasso regression confirmed this result (Burke Quinlan et al., 2015).

However, at present, DTI research in cerebral infarction disease is still in the initial stage, mostly limited to small sample sizes and short- or medium-term research, thus lacking large sample sizes and long-term studies. Moreover, the data from related animal trials and clinical trials are still very limited. Therefore, some challenges remain in using a single CST indicator to predict the recovery of motor function in clinical applications.

## BDNF Polymorphisms Affect Motor Recovery

Cerebral infarction is a class of diseases in which brain-tissue ischemia and hypoxia cause cellular metabolic disorders, ultimately leading to neuronal degeneration and death. It has been shown that endogenous BDNF and its receptor, tyrosine kinase (Trk) B, are increased under conditions of pathological change after stroke (Endres et al., 2000). BDNF is a member of the neurotrophin family and the most abundant neurotrophin in the body. It is synthesized in the central and peripheral nervous systems and is important in neuronal survival, differentiation, metabolism, damage repair, and regeneration (McTigue et al., 1998). BDNF promotes the repair and regeneration of neurons, accelerates the release of synaptic transmitters, strengthens synaptic transduction, promotes axonal growth, and promotes sensory neuron development, all of great value in the play of nerve-cell function (Lu et al., 2003; Fletcher et al., 2018). Therefore, BDNF plays a key role in neuroplasticity and motor recovery after subcortical stroke. It has been reported that local cerebral ischemia and hypoxia in acute stroke increase the expression of BDNF-specific receptors to protect nerve cells from damage (Tejeda et al., 2019). These studies demonstrate that BDNF levels can be used as an important indicator of treatment effect and prognosis after subcortical stroke.

Furthermore, it has been shown that BDNF can cross the blood-brain barrier; BDNF levels in the serum can thus reflect the level of BDNF in the brain. Testing the serum BDNF levels of patients can objectively and effectively reveal the severity of acute stroke. Early studies reported that an increase in serum BDNF levels correlated with motor recovery (Luo et al., 2019). Salinas et al. (2017) found that lower serum BDNF levels were associated with an increased risk of incident stroke. Some studies have also found that reduced BDNF levels are associated with severe functional impairment during the acute phase of ischemic stroke (Lasek-Bal et al., 2015; Stanne et al., 2016). Further studies also showed a reverse relationship between brain tissue damage and BDNF levels in the serum. For example, Qiao et al. (2017) found that BDNF levels decreased with increased infarct volumes; that is, the lower the level of BDNF, the higher the degree of damage to brain tissue. This showed that BDNF was protective against cerebral infarction and involved in the occurrence and development of cerebral infarction. In addition, greater levels of BDNF are associated with lesser white matter hyperintensity and better visual memory (Pikula et al., 2013). Serum BDNF has also been associated with post-stroke depression, and low serum BDNF levels may indicate the development of depression in patients with acute ischemic stroke (Yang et al., 2011).

BDNF is widely expressed in the central nervous system (CNS) and plays an important role in neural differentiation and synaptic plasticity (Binder and Scharfman, 2004). Moreover, BDNF polymorphisms affect the process of neuroplasticity by affecting the expression levels of neurotrophic factors in the brain (Stewart and Cramer, 2017). Several studies have shown that the single-nucleotide polymorphism (SNP) val^66^met (G189A or rs6265) of BDNF (Akbarian et al., 2018) is associated with stroke recovery (Shiner et al., 2016). Compared with stroke patients with the Val/Val gene, those with the Met gene had lower motor scores in the acute phase (7 days after stroke). However, the difference was not statistically significant 1 month after stroke (Mirowska-Guzel et al., 2012). Kim et al. (2012) found that stroke patients with the Met gene had lower motor scores than did those with the Val/Val gene in the subacute phase (4 months after stroke) but did not find similar results at 2 months after stroke. Some current findings have found that val^66^met SNPs do not predict long-term functional outcomes in stroke patients (French et al., 2018). Liepert et al. (2015) observed 67 patients with ischemic stroke, and their results indicated that polymorphic BDNF was closely related to the recovery of function after ischemic stroke. These findings indicate that BDNF contributes to the recovery and maintenance of brain function following stroke.

Animal experiments can provide a deeper understanding of complex physiological and pathological phenomena. Animal studies have shown that BDNF levels dynamically change, gradually increasing after birth, reaching a stable level in adulthood (Silhol et al., 2005), and gradually decreasing with advancing age (Wang et al., 2019). In a rat cerebral infarction model, BDNF and TrkB were permanently reduced at the center of cerebral infarcts, while the immune response in the ischemic penumbra was increased (Ferrer et al., 2001). Some researchers have found that the neuronal survival rate decreases significantly when BDNF activity is inhibited by the TrkB-Fc fusion protein (Larsson et al., 1999). These studies suggest that endogenous BDNF may protect neurons from ischemic damage and function as an endogenous neuroprotective agent. The neuroprotective role of BDNF in stroke has been demonstrated in subsequent intervention studies using exogenous BDNF. Zhang and Pardridge (2001) treated a mouse model of cerebral infarction with exogenous BDNF and observed reduced infarct volume at both 24 h and 7 days after treatment vs. control groups. Kiprianova et al. (1999) showed that intravenous infusion of BDNF in a continuous mouse model of stroke prevented the death of hippocampal CA1 neurons. Jiang et al. (2011) found no significant reduction in infarct volume in a mouse model of stroke after nasal BDNF administration but did find improvement in neurological recovery compared with control groups. These findings confirm that exogenous BDNF is meaningful in the treatment of cerebral infarction and revealed that BDNF also plays an active role in the treatment of stroke. In addition, rat models with knockout of the BDNF val^66^met single-nucleotide homolog (*BDNF^met/met^*) showed phenotypic characteristics similar o hose of humans (Chen et al., 2006). Compared with wild-type mice, *BDNF^met/met^* mice exhibited severe motor impairment only at 7 days after stroke, but not at 2 weeks or 1 month (Qin et al., 2011). Additionally, *BDNF^met/met^* mice showed reduced angiogenesis and elevated expression of thrombospondin-1 (TSP-1) and its receptor CD36, which are antiangiogenic factors. It has been suggested that the *Met* allele is associated with angiogenic deficits after stroke (Qin et al., 2011). These findings indicate that the relationship between BDNF polymorphisms and the degree of motor rehabilitation is complex and dependent on post-stroke stage. Therefore, combining brain microstructure findings with those from genetics can predict motor rehabilitation in stroke patients better than using a single index.

## Relationship Among CST Integrity, Genetics, and Prediction Models of Motor Recovery

From what has been discussed above, both CST integrity and BDNF genotype were shown to significantly influence to motor recovery of patients with stroke. Exploring the main factors that affect the efficacy of high-frequency repetitive transcranial magnetic stimulation to improve motor function in subacute stroke patients with moderate to severe upper extremity motor involvement, Chang et al. (2016) also found that the functional integrity of CST and BDNF genotype had the greatest impact on the improvement of upper extremity motor recovery. As well as, BDNF levels play a key role in white-matter microstructural plasticity (Lu et al., 2005; Jickling and Sharp, 2015). Luo et al. (2019) found that serum BDNF was positively correlated with FA in the CST in stroke patients with good motor recovery, whereas no such results were found in stroke patients with poor motor recovery. Park et al. found that in patients with stroke, FA in the ipsilesional CST was positively correlated with the motor outcome at 3 months in the presence of the Met genotype. These researchers concluded that the microstructural integrity of the intra-hemispheric tracts might be related to different processes of motor recovery dependent on the BDNF genotype (Park et al., 2020). Kim et al. (2016) also showed that poorer motor function was associated with higher radial diffusivity values in the CST for the *Val/Met* and *Met/Met* genotype groups in the early chronic stroke phase, which demonstrated that motor recovery in stroke patients might be affected by the BDNF val^66^met polymorphism, possibly through its effects on the distinct pathological processes underlying CST degeneration. In general, direct and/or indirect relationships between microstructure and genes are suggested by these findings, but causal relationships of these two factors have not been established. Exploring the microstructure of the CST as a potential mediator between gene expression and motor recovery is an urgent priority for understanding the rehabilitation mechanisms operating after subcortical stroke.

Few studies have combined data on CST microstructure with data on BDNF polymorphisms for the prediction of motor rehabilitation after stroke. Chang et al. predicted motor outcomes at 3 months using patient characteristics, integrity of the CST, and BDNF genotype. They found that in all stroke patients, the independent predictors of motor outcome at 3 months were baseline upper-extremity motor impairment, age, stroke type, and CST integrity. Further, in the group with severe motor impairment at baseline, the number of *Met* alleles in the BDNF genotype was an independent predictor of stroke (Chang et al., 2017). These results indicate that in patients with subacute stroke, the prediction of post-stroke motor recovery using CST integrity could be improved by the addition of the BDNF genotype factor. Therefore, prediction models combining CST with BDNF genotype are better than models with only one factor for stroke patients with severe motor impairment. Although using this combination strategy in patients at different stroke stages requires further validation in clinical applications, this idea provides new perspectives on how to establish a prediction model of post-stroke motor rehabilitation in the future.

## Conclusions

The microstructure of the CST and BDNF polymorphism status plays a key role in motor recovery after subcortical stroke. Future predictive models of stroke recovery are likely to include a combination of structural and genetic factors to determine patient-specific interventions for rehabilitation. These two factors are dynamic changes across different stages of stroke rehabilitation and may change depending on the amount of rehabilitation in patients with subcortical stroke. Moreover, their relationships and their predictive effect on stroke rehabilitation remain unclear. Linear mixed-effects model could be used to investigate the evolution of these two factors in a longitudinal dataset. Therefore, future studies should focus on verifying the roles of microstructure and genetic polymorphisms in motor rehabilitation in longitudinal designs with large samples, as well as on gene-knockout effects in mouse models of stroke to explore the relevant neurological mechanisms. These approaches will help to realize personalized rehabilitation strategies for stroke patients.

## Author Contributions

JL and CW contributed to conception and design of the study. JL wrote the first draft. All authors contributed to the article and approved the submitted version.

## Conflict of Interest

The authors declare that the research was conducted in the absence of any commercial or financial relationships that could be construed as a potential conflict of interest.

## Publisher’s Note

All claims expressed in this article are solely those of the authors and do not necessarily represent those of their affiliated organizations, or those of the publisher, the editors and the reviewers. Any product that may be evaluated in this article, or claim that may be made by its manufacturer, is not guaranteed or endorsed by the publisher.

## References

[B1] Akbarian S. A. Salehi-Abargouei A. Pourmasoumi M. Kelishadi R. Nikpour P. Heidari-Beni M. (2018). Association of Brain-derived neurotrophic factor gene polymorphisms with body mass index: a systematic review and meta-analysis. Adv. Med. Sci. 63, 43–56. 10.1016/j.advms.2017.07.002 28818748

[B2] Balkaya M. Cho S. (2019). Genetics of stroke recovery: BDNF val66met polymorphism in stroke recovery and its interaction with aging. Neurobiol. Dis. 126, 36–46. 10.1016/j.nbd.2018.08.009 30118755 PMC7653673

[B3] Berretta A. Tzeng Y. C. Clarkson A. N. (2014). Post-stroke recovery: the role of activity-dependent release of brain-derived neurotrophic factor. Expert Rev. Neurother. 14, 1335–1344. 10.1586/14737175.2014.969242 25319267

[B4] Binder D. K. Scharfman H. E. (2004). Brain-derived neurotrophic factor. Growth Factors 22, 123–131. 10.1080/08977190410001723308 15518235 PMC2504526

[B5] Burke Quinlan E. Dodakian L. See J. McKenzie A. Le V. Wojnowicz M.. (2015). Neural function, injury and stroke subtype predict treatment gains after stroke. Ann. Neurol. 77, 132–145. 10.1002/ana.24309 25382315 PMC4293339

[B6] Chang W. H. Park E. Lee J. Lee A. Kim Y. H. (2017). Association between brain-derived neurotrophic factor genotype and upper extremity motor outcome after stroke. Stroke 48, 1457–1462. 10.1161/STROKEAHA.116.015264 28495829

[B7] Chang W. H. Uhm K. E. Shin Y. I. Pascual-Leone A. Kim Y. H. (2016). Factors influencing the response to high-frequency repetitive transcranial magnetic stimulation in patients with subacute stroke. Restor. Neurol. Neurosci. 34, 747–755. 10.3233/RNN-150634 27372515

[B8] Chen Z. Y. Jing D. Bath K. G. Ieraci A. Khan T. Siao C. J.. (2006). Genetic variant BDNF (Val66Met) polymorphism alters anxiety-related behavior. Science 314, 140–143. 10.1126/science.1129663 17023662 PMC1880880

[B9] Endres M. Fan G. Hirt L. Fujii M. Matsushita K. Liu X.. (2000). Ischemic brain damage in mice after selectively modifying BDNF or NT4 gene expression. J. Cereb. Blood Flow Metab. 20, 139–144. 10.1097/00004647-200001000-00018 10616802

[B10] Ferrer I. Krupinski J. Goutan E. Marti E. Ambrosio S. Arenas E. (2001). Brain-derived neurotrophic factor reduces cortical cell death by ischemia after middle cerebral artery occlusion in the rat. Acta Neuropathol. 101, 229–238. 10.1007/s004010000268 11307622

[B11] Fletcher J. L. Murray S. S. Xiao J. (2018). Brain-derived neurotrophic factor in central nervous system myelination: a new mechanism to promote myelin plasticity and repair. Int. J. Mol. Sci. 19:4131. 10.3390/ijms19124131 30572673 PMC6321406

[B12] French M. A. Morton S. M. Pohlig R. T. Reisman D. S. (2018). The relationship between BDNF Val66Met polymorphism and functional mobility in chronic stroke survivors. Top. Stroke Rehabil. 25, 276–280. 10.1080/10749357.2018.1437938 29480080 PMC5901741

[B13] Guo J. Liu J. Wang C. Cao C. Fu L. Han T.. (2019). Differential involvement of rubral branches in chronic capsular and pontine stroke. Neuroimage Clin. 24:102090. 10.1016/j.nicl.2019.102090 31835285 PMC6911903

[B14] Jiang Y. Wei N. Lu T. Zhu J. Xu G. Liu X. (2011). Intranasal brain-derived neurotrophic factor protects brain from ischemic insult *via* modulating local inflammation in rats. Neuroscience 172, 398–405. 10.1016/j.neuroscience.2010.10.054 21034794

[B15] Jickling G. C. Sharp F. R. (2015). Biomarker panels in ischemic stroke. Stroke 46, 915–920. 10.1161/STROKEAHA.114.005604 25657186 PMC4342265

[B17] Johnson B. P. Westlake K. P. (2021). Chronic poststroke deficits in gross and fine motor control of the ipsilesional upper limb. Am. J. Phys. Med. Rehabil. 100, 345–348. 10.1097/PHM.0000000000001569 32804714 PMC12404851

[B19] Kim H. Lee H. Jung K. I. Ohn S. H. Yoo W. K. (2018). Changes in diffusion metrics of the red nucleus in chronic stroke patients with severe corticospinal tract injury: a preliminary study. Ann. Rehabil. Med. 42, 396–405. 10.5535/arm.2018.42.3.396 29961737 PMC6058581

[B18] Kim E. J. Park C. H. Chang W. H. Lee A. Kim S. T. Shin Y. I.. (2016). The brain-derived neurotrophic factor Val66Met polymorphism and degeneration of the corticospinal tract after stroke: a diffusion tensor imaging study. Eur. J. Neurol. 23, 76–84. 10.1111/ene.12791 26228236

[B16] Kim B. Schweighofer N. Haldar J. P. Leahy R. M. Winstein C. J. (2021). Corticospinal tract microstructure predicts distal arm motor improvements in chronic stroke. J. Neurol. Phys. Ther. 45, 273–281. 10.1097/NPT.0000000000000363 34269747 PMC8460613

[B20] Kim J. M. Stewart R. Park M. S. Kang H. J. Kim S. W. Shin I. S.. (2012). Associations of BDNF genotype and promoter methylation with acute and long-term stroke outcomes in an East Asian cohort. PLoS One 7:e51280. 10.1371/journal.pone.0051280 23240009 PMC3519835

[B21] Kiprianova I. Freiman T. M. Desiderato S. Schwab S. Galmbacher R. Gillardon F.. (1999). Brain-derived neurotrophic factor prevents neuronal death and glial activation after global ischemia in the rat. J. Neurosci. Res. 56, 21–27. 10.1002/(SICI)1097-4547(19990401)56:1<21::AID-JNR3>3.0.CO;2-Q 10213471

[B22] Larsson E. Nanobashvili A. Kokaia Z. Lindvall O. (1999). Evidence for neuroprotective effects of endogenous brain-derived neurotrophic factor after global forebrain ischemia in rats. J. Cereb. Blood Flow Metab. 19, 1220–1228. 10.1097/00004647-199911000-00006 10566968

[B23] Lasek-Bal A. Jedrzejowska-Szypulka H. Rozycka J. Bal W. Holecki M. Dulawa J.. (2015). Low concentration of BDNF in the acute phase of ischemic stroke as a factor in poor prognosis in terms of functional status of patients. Med. Sci. Monit. 21, 3900–3905. 10.12659/msm.895358 26656843 PMC4684138

[B24] Liepert J. Heller A. Behnisch G. Schoenfeld A. (2015). [Polymorphism of brain derived neurotrophic factor and recovery of functions after ischemic stroke]. Nervenarzt 86, 1255–1260. 10.1007/s00115-015-4325-6 26391958

[B25] Lin D. J. Cloutier A. M. Erler K. S. Cassidy J. M. Snider S. B. Ranford J.. (2019). Corticospinal tract injury estimated from acute stroke imaging predicts upper extremity motor recovery after stroke. Stroke 50, 3569–3577. 10.1161/STROKEAHA.119.025898 31648631 PMC6878199

[B26] Liu J. Qin W. Zhang J. Zhang X. Yu C. (2015). Enhanced interhemispheric functional connectivity compensates for anatomical connection damages in subcortical stroke. Stroke 46, 1045–1051. 10.1161/STROKEAHA.114.007044 25721013

[B27] Liu J. Wang C. Qin W. Ding H. Guo J. Han T.. (2020). Corticospinal fibers with different origins impact motor outcome and brain after subcortical stroke. Stroke 51, 2170–2178. 10.1161/STROKEAHA.120.029508 32568657

[B29] Lu D. Mahmood A. Chopp M. (2003). Biologic transplantation and neurotrophin-induced neuroplasticity after traumatic brain injury. J. Head Trauma Rehabil. 18, 357–376. 10.1097/00001199-200307000-00006 16222130

[B28] Lu B. Pang P. T. Woo N. H. (2005). The yin and yang of neurotrophin action. Nat. Rev. Neurosci. 6, 603–614. 10.1038/nrn1726 16062169

[B30] Luo W. Liu T. Li S. Wen H. Zhou F. Zafonte R.. (2019). The serum BDNF level offers minimum predictive value for motor function recovery after stroke. Transl. Stroke Res. 10, 342–351. 10.1007/s12975-018-0648-5 30074228 PMC6391224

[B31] McTigue D. M. Horner P. J. Stokes B. T. Gage F. H. (1998). Neurotrophin-3 and brain-derived neurotrophic factor induce oligodendrocyte proliferation and myelination of regenerating axons in the contused adult rat spinal cord. J. Neurosci. 18, 5354–5365. 10.1523/JNEUROSCI.18-14-05354.1998 9651218 PMC6793495

[B32] Mirowska-Guzel D. Gromadzka G. Czlonkowski A. Czlonkowska A. (2012). BDNF -270 C>T polymorphisms might be associated with stroke type and BDNF -196 G>A corresponds to early neurological deficit in hemorrhagic stroke. J. Neuroimmunol. 249, 71–75. 10.1016/j.jneuroim.2012.04.011 22633192

[B33] Mori S. van Zijl P. C. (2002). Fiber tracking: principles and strategies - a technical review. NMR Biomed. 15, 468–480. 10.1002/nbm.781 12489096

[B34] Newton J. M. Ward N. S. Parker G. J. Deichmann R. Alexander D. C. Friston K. J.. (2006). Non-invasive mapping of corticofugal fibres from multiple motor areas—relevance to stroke recovery. Brain 129, 1844–1858. 10.1093/brain/awl106 16702192 PMC3718077

[B35] Park E. Lee J. Chang W. H. Lee A. Hummel F. C. Kim Y. H. (2020). Differential relationship between microstructural integrity in white matter tracts and motor recovery following stroke based on brain-derived neurotrophic factor genotype. Neural Plast. 2020:5742421. 10.1155/2020/5742421 33029116 PMC7527931

[B36] Patel P. Kaingade S. R. Wilcox A. Lodha N. (2020). Force control predicts fine motor dexterity in high-functioning stroke survivors. Neurosci. Lett. 729:135015. 10.1016/j.neulet.2020.135015 32360934

[B37] Pikula A. Beiser A. S. Chen T. C. Preis S. R. Vorgias D. DeCarli C.. (2013). Serum brain-derived neurotrophic factor and vascular endothelial growth factor levels are associated with risk of stroke and vascular brain injury: framingham study. Stroke 44, 2768–2775. 10.1161/STROKEAHA.113.001447 23929745 PMC3873715

[B38] Qiao H. J. Li Z. Z. Wang L. M. Sun W. Yu J. C. Wang B. (2017). Association of lower serum Brain-derived neurotrophic factor levels with larger infarct volumes in acute ischemic stroke. J. Neuroimmunol. 307, 69–73. 10.1016/j.jneuroim.2017.04.002 28495141

[B39] Qin L. Kim E. Ratan R. Lee F. S. Cho S. (2011). Genetic variant of BDNF (Val66Met) polymorphism attenuates stroke-induced angiogenic responses by enhancing anti-angiogenic mediator CD36 expression. J. Neurosci. 31, 775–783. 10.1523/JNEUROSCI.4547-10.2011 21228186 PMC3308129

[B40] Radlinska B. Ghinani S. Leppert I. R. Minuk J. Pike G. B. Thiel A. (2010). Diffusion tensor imaging, permanent pyramidal tract damage and outcome in subcortical stroke. Neurology 75, 1048–1054. 10.1212/WNL.0b013e3181f39aa0 20855848 PMC2942063

[B41] Riley J. D. Le V. Der-Yeghiaian L. See J. Newton J. M. Ward N. S.. (2011). Anatomy of stroke injury predicts gains from therapy. Stroke 42, 421–426. 10.1161/STROKEAHA.110.599340 21164128 PMC3026869

[B42] Salinas J. Beiser A. Himali J. J. Satizabal C. L. Aparicio H. J. Weinstein G.. (2017). Associations between social relationship measures, serum brain-derived neurotrophic factor and risk of stroke and dementia. Alzheimers Dement. (N Y) 3, 229–237. 10.1016/j.trci.2017.03.001 29067329 PMC5651441

[B43] Schaechter J. D. Fricker Z. P. Perdue K. L. Helmer K. G. Vangel M. G. Greve D. N.. (2009). Microstructural status of ipsilesional and contralesional corticospinal tract correlates with motor skill in chronic stroke patients. Hum. Brain Mapp. 30, 3461–3474. 10.1002/hbm.20770 19370766 PMC2780023

[B44] Schieber M. H. (2007). Chapter 2 Comparative anatomy and physiology of the corticospinal system. Handb. Clin. Neurol. 82, 15–37. 10.1016/S0072-9752(07)80005-4 18808887

[B45] Seo J. P. Jang S. H. (2013). Different characteristics of the corticospinal tract according to the cerebral origin: DTI study. Am. J. Neuroradiol. 34, 1359–1363. 10.3174/ajnr.A3389 23370470 PMC8051479

[B46] Shiner C. T. Pierce K. D. Thompson-Butel A. G. Trinh T. Schofield P. R. McNulty P. A. (2016). BDNF genotype interacts with motor function to influence rehabilitation responsiveness poststroke. Front. Neurol. 7:69. 10.3389/fneur.2016.00069 27242654 PMC4868962

[B47] Silhol M. Bonnichon V. Rage F. Tapia-Arancibia L. (2005). Age-related changes in brain-derived neurotrophic factor and tyrosine kinase receptor isoforms in the hippocampus and hypothalamus in male rats. Neuroscience 132, 613–624. 10.1016/j.neuroscience.2005.01.008 15837123

[B48] Stanne T. M. Aberg N. D. Nilsson S. Jood K. Blomstrand C. Andreasson U.. (2016). Low circulating acute brain-derived neurotrophic factor levels are associated with poor long-term functional outcome after ischemic stroke. Stroke 47, 1943–1945. 10.1161/STROKEAHA.115.012383 27301948

[B49] Sterr A. Dean P. J. Szameitat A. J. Conforto A. B. Shen S. (2014). Corticospinal tract integrity and lesion volume play different roles in chronic hemiparesis and its improvement through motor practice. Neurorehabil. Neural Repair 28, 335–343. 10.1177/1545968313510972 24334657

[B50] Stewart J. C. Cramer S. C. (2017). Genetic variation and neuroplasticity: role in rehabilitation after stroke. J. Neurol. Phys. Ther. 41, S17–S23. 10.1097/NPT.0000000000000180 28628592 PMC5477674

[B51] Stinear C. M. Barber P. A. Smale P. R. Coxon J. P. Fleming M. K. Byblow W. D. (2007). Functional potential in chronic stroke patients depends on corticospinal tract integrity. Brain 130, 170–180. 10.1093/brain/awl333 17148468

[B52] Tejeda G. S. Esteban-Ortega G. M. San Antonio E. Vidaurre O. G. Diaz-Guerra M. (2019). Prevention of excitotoxicity-induced processing of BDNF receptor TrkB-FL leads to stroke neuroprotection. EMBO Mol. Med. 11:e9950. 10.15252/emmm.201809950 31273936 PMC6609917

[B53] Wang G. Zhou Y. Wang Y. Li D. Liu J. Zhang F. (2019). Age-associated dopaminergic neuron loss and midbrain glia cell phenotypic polarization. Neuroscience 415, 89–96. 10.1016/j.neuroscience.2019.07.021 31325563

[B54] Welniarz Q. Dusart I. Roze E. (2017). The corticospinal tract: evolution, development and human disorders. Dev. Neurobiol. 77, 810–829. 10.1002/dneu.22455 27706924

[B55] Xue Q. Yang X. H. Teng G. J. Hu S. D. (2021). Chronic pontine strokes: diffusion tensor imaging of corticospinal tract indicates the prognosis in terms of motor outcome. J. Xray Sci. Technol. 29, 477–489. 10.3233/XST-200817 33720869

[B56] Yang L. Zhang Z. Sun D. Xu Z. Yuan Y. Zhang X.. (2011). Low serum BDNF may indicate the development of PSD in patients with acute ischemic stroke. Int. J. Geriatr. Psychiatry 26, 495–502. 10.1002/gps.2552 20845405

[B57] Yu C. Zhu C. Zhang Y. Chen H. Qin W. Wang M.. (2009). A longitudinal diffusion tensor imaging study on Wallerian degeneration of corticospinal tract after motor pathway stroke. Neuroimage 47, 451–458. 10.1016/j.neuroimage.2009.04.066 19409500

[B58] Zhang Y. Pardridge W. M. (2001). Neuroprotection in transient focal brain ischemia after delayed intravenous administration of brain-derived neurotrophic factor conjugated to a blood-brain barrier drug targeting system. Stroke 32, 1378–1384. 10.1161/01.str.32.6.1378 11387502

[B59] Zhu L. L. Lindenberg R. Alexander M. P. Schlaug G. (2010). Lesion load of the corticospinal tract predicts motor impairment in chronic stroke. Stroke 41, 910–915. 10.1161/STROKEAHA.109.577023 20378864 PMC2886713

[B60] Zolkefley M. K. I. Firwana Y. M. S. Hatta H. Z. M. Rowbin C. Nassir C. Hanafi M. H.. (2021). An overview of fractional anisotropy as a reliable quantitative measurement for the corticospinal tract (CST) integrity in correlation with a Fugl-Meyer assessment in stroke rehabilitation. J. Phys. Ther. Sci. 33, 75–83. 10.1589/jpts.33.75 33519079 PMC7829559

